# A Family-Centered Sexual Health Intervention to Promote Cervical Cancer Screening Uptake Among Low-Income Rural Women in India: Protocol for a Community-Based Mixed Methods Pilot Study

**DOI:** 10.2196/35093

**Published:** 2022-09-08

**Authors:** Mandana Vahabi, Aisha K Lofters, Gauravi Mishra, Sharmila Pimple, Josephine Pui-Hing Wong

**Affiliations:** 1 Daphne Cockwell School of Nursing Toronto Metropolitan University (formerly known as Ryerson University) Toronto, ON Canada; 2 Women’s College Hospital Toronto, ON Canada; 3 Tata Memorial Centre Maharashtra India

**Keywords:** cervical cancer screening, human papillomavirus self-sampling, India, low income, sexual health, health literacy, women, family-centered care, rural area, rural, sexual health literacy, human papillomavirus, screening, cancer screening, cervical, cancer, sexually transmitted infection

## Abstract

**Background:**

Human papillomavirus (HPV) is the primary cause of cervical cancer, which is preventable through screening and early treatment. The Papanicolaou (Pap) test and visual inspection with acetic acid (VIA), which are traditionally performed in clinical settings, have been used effectively to screen for cervical cancer and precancerous changes and reduce cervical cancer mortality in high-income countries for many decades. However, these screening methods are not easily accessible to women living in low- and middle-income countries, especially women living in rural areas.

**Objective:**

The project will use HPV self-sampling, which will be supported by a sexual health literacy intervention, to increase rural women’s participation in cervical cancer screening. The objectives are to determine the effectiveness of this program in (1) increasing sexual health literacy, (2) reducing the gendered stigma of HPV and cervical cancer, and (3) promoting cervical cancer screening by using HPV self-sampling.

**Methods:**

The pilot study will use a community-based, family-centered, mixed methods design. We will recruit 120 women aged 30 to 69 years who are underscreened or were never screened for cervical cancer, along with 120 supportive male relatives or friends from 3 low-income rural/tribal villages in Maharashtra, India. Participants will attend gender-specific sexual health education sessions, followed by a movie matinee. Data will be collected through an interviewer-administered questionnaire before and after sexual health education sessions. The questionnaire will include items on social demographics, medical histories, attitudes, sexual health stigma, cervical cancer knowledge, and screening practices. Women will self-select whether to use HPV self-sampling. Those who do not may undergo a Pap test or VIA. Participants’ views regarding barriers and facilitators and their suggestions for improving access and uptake will also be elicited. This protocol was approved by the research ethics boards of Toronto Metropolitan University (formerly known as Ryerson University; reference number: REB 2020-104) and Tata Memorial Center (reference number: OIEC/3786/2021 /00003).

**Results:**

The Preventing Cervical Cancer in India Through Self-Sampling study was funded in January 2020 for 15 months. Due to the COVID-19 pandemic, the project was extended by 1 year. The study outcome measures will include changes in knowledge and attitudes about cervical cancer screening, the proportion of participants who self-select into each cohort, the proportion of positive test results in each cohort, and the proportion of participants with confirmed cervical cancer. Women’s experiences regarding barriers and facilitators of screening uptake will be captured.

**Conclusions:**

Our multifaceted work could lead to reduced cervical cancer mortality and morbidity and increased community capacity in sexual health promotion and cervical cancer prevention. The insights and lessons learned from our project can be used to inform the adaptation and scale-up of HPV self-sampling among women across India and in other countries; promote collective commitment to family-centered wellness; and support women to make healthful, personalized cervical screening decisions.

**International Registered Report Identifier (IRRID):**

PRR1-10.2196/35093

## Introduction

Cervical cancer is a vivid indicator of global health disparity, as 90% of all deaths due to cervical cancer occur in low- and middle-income countries [1]. It is one of the leading causes of cancer-related mortality worldwide, and India accounts for 16% of cervical cancer cases and 30% of cervical cancer deaths globally [1-4]. Every year in India, 96,922 new cervical cancer cases are diagnosed, and 60,078 women die due to late diagnosis and no access to lifesaving treatment [2,4]. It is the second leading cause of cancer death in women aged 15 to 44 years in India [2]. Virtually all cases of cervical cancer are caused by human papillomavirus (HPV), which is transmitted primarily through sexual intercourse. Cervical cancer has profound negative impacts on individual life expectancy, the quality of family life, and personal and societal economic burden. However, premature death due to cervical cancer and disability resulting from cervical cancer are preventable tragedies that can be avoided through regular screening. With appropriate screening, cervical cancer is highly preventable. In many high-income nations, the incidence and mortality of cervical cancer have declined steadily and steeply as a result of the widespread use of the Papanicolaou (Pap) test as a screening tool. However, the coverage of cervical cancer screening is 19% in low- and middle-income countries and 63% in high-income countries [5]. Moreover, preadolescent HPV vaccination is too expensive for widespread use in low-income countries, given its social, logistical, and financial challenges.

Cervical cancer screening and testing for the HPV viruses are available at urban private medical centers in India but not in government health centers. The burden of preventing cervical cancer is solely carried by the Indian women themselves, with little support or resources. Women in rural India are particularly vulnerable because they often have no knowledge about screening or have no access to screening services, and many have low literacy and no access to education and resources, including financial resources [6]. Furthermore, many of them get married at ages as young as 15 years, even though the legal age of marriage in India is 18 years [7], meaning that they become sexually active at a young age. They are also often socially and economically dependent on the men in their families [8], are often excluded from decision-making, and are often restricted in terms of mobility [9]. Studies that have been conducted with women in India and among the South Asian diaspora in high-income countries have found limited knowledge about cervical cancer and the benefits of screening among the participants [10,11]. Furthermore, misconceptions and stigmas surrounding sexually transmitted infections (STIs), including HPV, may deter the uptake of screening due to the entrenched gender norms and stereotypes associated with infections (eg, women with STIs may be viewed as immoral or corrupt, among other demeaning labels). Engaging both men and women in cervical cancer screening and normalizing, destigmatizing, and talking openly about cervical cancer and screening are important steps in reducing cervical cancer mortality and morbidity. Male partners’ involvement in sexual and preventive health care has been shown to not only be an effective strategy in women’s health outcomes but also be acceptable to women in general [12-16]. The World Health Organization recommends men’s engagement in the prevention of cervical cancer in low- and middle-income countries [16]. Men play a significant role in their female partners’ access to health services, as they are the main source of financial support for costs related to transportation and health services. Moreover, they hold the primary power in granting permission for using these health services. Thus, it is imperative to provide alternative, accessible, acceptable, innovative, and family-centered screening strategies that could help reduce observed cervical cancer disparities.

HPV self-sampling is an easy and user-friendly method that has been shown to be highly acceptable among women in diverse racial, ethnic, religious, and age groups in many countries [17-31]. This alternative approach has been found to be effective in engaging underscreened and never-screened (UNS) women to increase their participation in cervical cancer screening and reduce related mortality and morbidity, thereby improving the overall quality of life among women [18,19,21,22,25,27,28]. It does not require a health care provider to either perform a pelvic examination (whereas Pap tests do require a health care provider) or conduct a vaginal speculum examination that involves applying diluted acetic acid (vinegar) to the cervix. This means that there is no need for traveling to medical centers, which often is a barrier. HPV self-sampling provides an opportunity for empowering women by allowing them to conduct such tests in the privacy of their own homes and at a time that is convenient to them. Offering this approach as a screening alternative for UNS groups could lead to increased participation and a resultant reduction in cancer screening inequalities.

This paper presents the study protocol for an international study titled *Preventing Cervical Cancer in India Through Self-Sampling (PCCIS)*. This is a family-centered, community-based, mixed methods pilot project that aims to improve the health of rural Indian women residing in the state of Maharashtra through capacity building and by reducing avoidable health disparities that are associated with HPV and cervical cancer. A key focus of this project is to promote gender equity through collective empowerment and capacity building. Gender equity is achieved through increasing women’s access to lifesaving tests and health resources, enabling equitable decision-making, and changing attitudes by engaging both men and women as families. This family-centered approach will contribute to open dialogues about and solutions for reducing stigma and improving community health literacy with regard to HPV and cervical cancer screening and, consequently, will increase cervical cancer screening uptake.

## Methods

### Study Design

We will use a community-based mixed methods design to assess the effectiveness of a family- centered program that involves using HPV self-sampling, which will be supported by a sexual health literacy intervention, to increase rural women’s participation in HPV and cervical cancer screening. The study objectives are to determine the effectiveness of this program in (1) increasing sexual health literacy, (2) reducing the gendered stigma of HPV and cervical cancer, and (3) promoting cervical cancer screening by using HPV self-sampling.

### Target Population

We will engage 3 rural/tribal villages—Shirgoan in Phalghar district, Khodala in Mokhada district, and Jamsar in Jawhar district—in the state of Maharashtra, which is the second most populated state of India; Maharashtra has a population of over 128 million people and a high prevalence of advanced cervical cancer. Approximately 55% of Phalghar’s total population lives in rural areas. The literacy rate in Phalghar is about 67% (59% for women and 72% for men). However, the literacy rate drops significantly in the rural areas of this district. For example, the literacy level is reported to be 46% in Mokhada and 48% in Jawhar. The women in these areas are expected to have all of the risk factors pertaining to cervical cancer and reproductive tract infections. The primary health center has no organized cervical cancer screening program. The women in these villages need to travel about 50 km to access medical facilities [32,33].

### Participants and Recruitment Strategies

The women in the above-mentioned villages are the primary target population of our project, and supportive men in their families (eg, fathers, brothers, spouses, cousins, and sons) are the secondary target population. We will recruit 120 women and 120 men from the women’s families.

Our project will build capacity by training trusted female community members in these villages to act as community champions and help recruit and educate women about cervical cancer and the use of HPV self-sampling. The community champions will be *local* *accredited social health activists* (termed *ASHA*, meaning *hope*)—a new model of care that was adopted by the Indian government; it relies on building capacity in local and remote areas by recruiting trusted community members and training them to promote awareness about the health-related issues facing their respective communities. The community champions will assist with the participants' recruitment. Once the women agree to participate, they will be invited to identify a “supportive” man in their life (eg, a spouse, father, brother, cousin, or son) whom they feel can play a positive and influential role in health promotion within their villages.

The eligibility criteria for female participants include (1) UNS women (ie, a self-report of >4 years since their last Pap test or visual inspection with acetic acid [VIA], including no history of a Pap test or VIA); (2) women aged 30 to 69 years old; (3) women residing in Shirgoan, Khodala, and Jamsar in the state of Maharashtra, India; (4) women who have ever been sexually active; (5) women who are able to provide informed consent; and (6) women who are willing to share contact information with the study team. The exclusion criteria include (1) being pregnant and (2) having a history of hysterectomy or past treatment for precancerous or cancerous cervical lesions.

Male participants’ eligibility criteria include (1) being a male family member (husbands, fathers, brothers, sons, and cousins) of the UNS female participants, (2) being identified and referred by UNS female participants, and (3) being aged 18 years or older.

### Theoretical Framework

**Figure 1:**
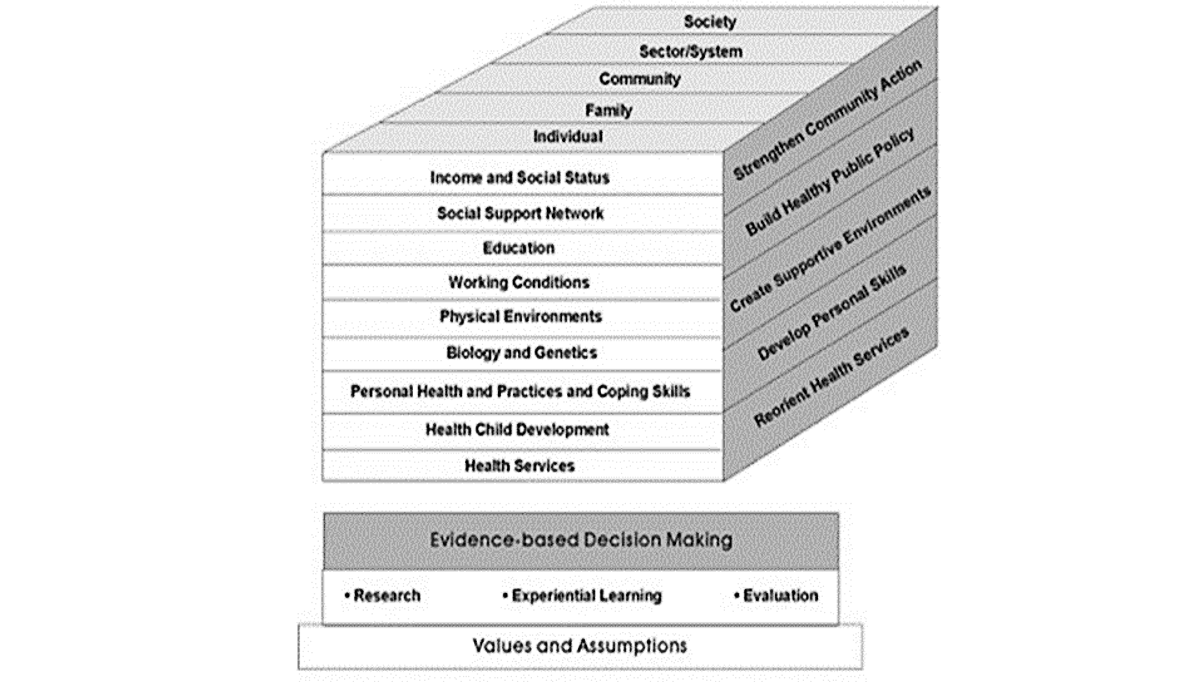
Population Health Promotion Model [34].

Our study will be guided by the Population Health Promotion framework [34] (Figure 1), which is grounded in the principles of social justice and equity and uses a socioenvironmental approach to address health disparities [35,36]. This research approach is aligned with the 2018 Whistler Principles to Accelerate Innovation for Development Impact [37]. Our research approach recognizes that individual and collective health are intertwined and that health disparities are the outcomes of intersecting social determinants, including access to economic and social resources, everyday encounters of gender-based discrimination, and social exclusion [38,39]. It is underpinned by the concepts of (1) community empowerment, a process that promotes the participation of people, organizations, and communities toward the goal of increased individual and community control, the improved quality of community life, and social justice, and (2) capacity building, in which existing human and social resources are leveraged to solve collective problems and improve the well-being of a community’s members through informal and formal social processes and organized efforts [35,38].

**Figure 2:**
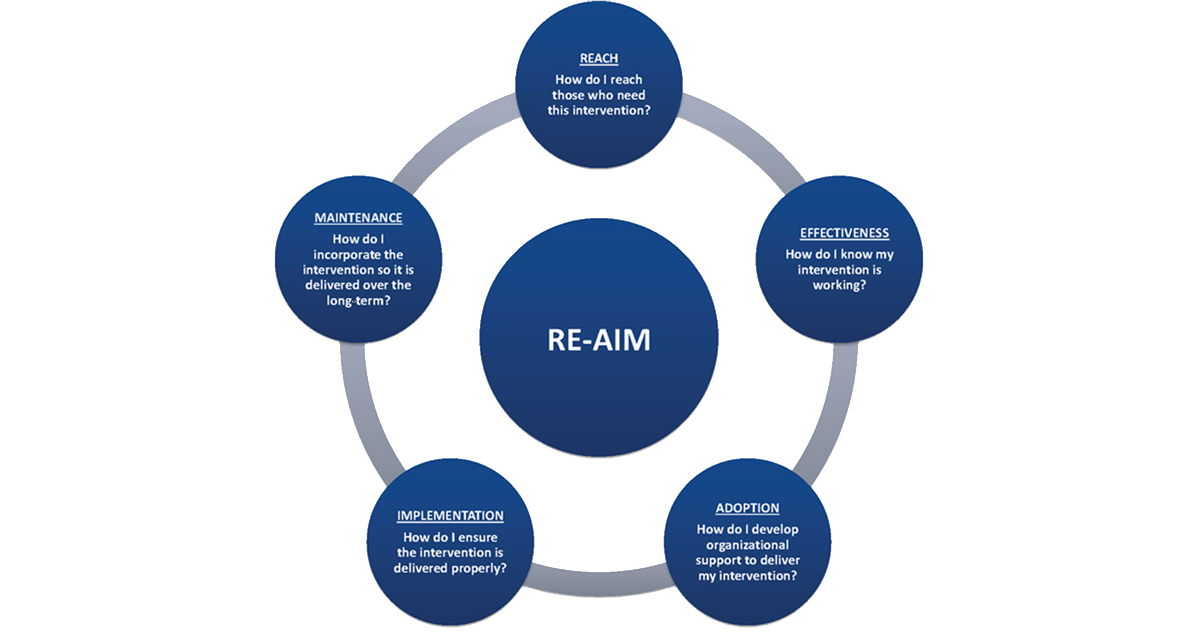
The RE-AIM framework. RE-AIM: Reach, Effectiveness, Adoption, Implementation, and Maintenance.

We will also use the Reach, Effectiveness, Adoption, Implementation, and Maintenance (RE-AIM) framework [40,41] (Figure 2) to evaluate the overall public health impact of our intervention. This framework allows for the comprehensive assessment of public health programs in a real-world setting. We will assess whether we are *reaching* our intended target of UNS rural Indian women and the supportive men in their lives; whether the intervention is *effective* by quantifying screening uptake, normal and abnormal screening test results, and their end results; how many women and providers *adopt* the intervention over the study period; how much training, education, and staff time are required in *implementation*; and whether the screening program is *maintained* after the study period.

### Study Intervention

The project is a community-based, action-oriented intervention with the following two components: education and access to cervical cancer screening. The central hypotheses that we will address are that (1) there will be an increase in sexual health literacy (knowledge) and a reduction in sexual health stigma (attitudes) related to cervical cancer and screening after participating in the study and (2) there will be a higher uptake of HPV self-sampling compared to that of traditional methods of screening (VIA and Pap test) among UNS women. To test these two hypotheses, we will begin with the education component, which will be followed by inviting women to take part in HPV self-sampling.

### Education to Promote Sexual Health and HPV Screening

The sexual health literacy intervention is family- and community-centered. The intervention activities will promote the awareness and sexual health literacy of both men and women in our three targeted villages. Strong evidence indicates that family- and community-centered approaches are needed to advance gender equity because (1) women and girls do not live and are not socialized in a vacuum, (2) decisions about every aspect of the lives of women and girls are embedded in their family relationships and intimate relationships, and (3) the stigma of HPV and cervical cancer is often reinforced by dominant society norms and misconceptions [42]. By investing in a program that increases sexual health knowledge among women, the men in their families, and their communities as a whole, we anticipate that the program’s impact will be sustainable.

Since the literacy level of the women and men in the three selected villages is low, we will use a storytelling health communication approach that has been proven to be effective in promoting adult health literacy [43,44]. Storytelling is an integral aspect of everyday life. Stories enable participants to engage with the content in ways that allow them to make sense of the content in the context of their own lives, such as through self-reflection and dialogue with others [45,46]. Furthermore, stories are effective in reducing stigma [38] and changing attitudes, as they carry the power to engage the audience emotively to promote empathy, shape perspectives, and motivate action [47,48]. Storytelling is a well-established and well-accepted means of cultural learning and empowerment in India [49,50]. It is also a strategy for sharing knowledge, particularly among women who have been historically left out of more formal learning institutions [51].

To ensure that the project activities are socially relevant and culturally appropriate, we will establish a project advisory committee (PAC) prior to implementing the project. The PAC will be made up of local community members (including both men and women), women advocates, service providers, decision makers, and relevant stakeholders. We will actively engage the PAC in all stages of the project to provide support for design refinement, community engagement, recruitment, the interpretation of results, and knowledge translation and exchange activities. With input from the PAC, we will develop culturally appropriate education materials and videos in Hindi and Marathi—the predominant languages spoken in Maharashtra. Storytelling will be used in the following three accessible formats to promote learning: graphic novels, short videos, and testimonials from service providers and health professionals. In addition, infographics and illustrations will be used to support women in learning how to perform HPV self-sampling, along with support from the community champions and health professionals. Together, these materials will promote knowledge about the HPV virus, cervical cancer, cervical screening, HPV self-sampling, sexual health, and general health as family resources.

### Gender-Specific Sexual Health Education Sessions

We will hold 24 gender-specific sexual health education (SHE) sessions—12 for women and 12 for men. A male clinician will lead the men’s sessions, and a female clinician will lead the women’s sessions. Each information session will be approximately 90 to 120 minutes in length and consist of 10 participants. The topics will include the HPV virus in the context of STIs, the stigma surrounding cervical cancer, the method of HPV virus transmission, the male and female cancers that result from HPV infection, the risk factors of cervical cancer, and the importance of screening. Questions about knowledge, attitudes, and stigma will be adopted from validated questionnaires and will be reviewed by the PAC for cultural relevance and appropriateness. Other gender-specific content, which will be identified through consultations with the PAC and local team members, will be included to facilitate increased health literacy and promote participants’ interest.

All participants will then be invited to attend a movie matinee on the same day as the SHE. The contents of the video for the movie matinee will be drawn from clinical cases and will include stories of two women—one who died of cervical cancer (ie, a movie about how her death impacted her spouse and family) and another who was screened, was diagnosed, received treatment, and recovered. This video will be followed by short testimonial videos from health care professionals, those from affected women and family members, and a period in which the audience can ask questions and receive answers. Packed food and refreshments will be served at these sessions. The SHE sessions and the movie matinee will be held in a venue, and in accordance with the local COVID-19 guidelines, we will ensure proper social distancing and the wearing of masks and personal protective equipment by all attendees. All participants will complete an interviewer-administered questionnaire before and after the SHE sessions. At the end of the movie matinee, all participants will be surveyed on their intention to take part in HPV self-sampling. They will be given 3 colored paper ribbons with preprinted potential participation codes when they arrive (red=no; green=yes; orange=undecided), which will represent their family-centered decision to participate in HPV self-sampling. In other words, the decision to undertake screening will be made together by female participants and their selected male participants at the end of the movie matinee, so that only 1 color-coded ribbon will be selected by each couple and shared with the research staff.

### HPV Testing Process and Benchmarks

Based on the responses that we receive at the end of the movie matinee, female participants will be divided into 2 self-selected cohorts. Cohort A will consist of eligible women who are willing to use the HPV self-sampling kit. Cohort B will consist of eligible women who do not agree to use the kit but consent to participate in the study, and they may or may not undergo VIA or a Pap test.

Our Indian study collaborators and informants indicated that we should expect to recruit ≥100 women in cohort A and 20 in cohort B over the study period. Those who are interested in trying HPV self-sampling will be provided with a kit that they can take home and a visual instruction pamphlet that displays how to collect a sample. They will be told that a female health professional will pick up their self-collected specimen within 48 hours. The female health professional will then collect the specimens and take them to the designated hospital labs. Those who test negative for HPV will be informed by their respective female health professional, and their names will be included in a database that will be developed and maintained by our local partners, so that these participants can be contacted again for screening in 5 years. Those who test positive will undergo VIA or a Pap test in a designated community place. The tests will be performed by either our research health care providers or local gynecologists, who will be trained by our Indian study collaborators.

Medical follow-up tests and treatments will be arranged by our Indian study collaborators for participants with positive Pap test results. For women in cohort B who refuse HPV self-sampling but agree to undergo VIA or a Pap test, our medically trained female health professional will either perform VIAs or arrange their Pap tests through our medical collaborators. Those with negative VIA test or Pap test results will be informed by our health professional, and their names will be added to the database, so that they can be contacted in 3 years. Those with positive Pap test results will undergo follow-up tests and treatments, as described above.

### Key Outcome Indicators

Informed by the Population Health Promotion and the RE-AIM frameworks, we will monitor and measure the following key indicators:

- Screening outcomes (among those who participate): the number of women screened (through self-sampling vs through VIA or a Pap test), the number of HPV-positive women detected by each screening method, the number of HPV-positive women that followed up by undergoing a Pap test, and the number of Pap-tested women referred to colposcopy (follow-up)
- Acceptability and preference outcomes: the usability of the screening kit and instructions, confidence in the sample collected, comfort with and preference for self-sampling versus VIA and Pap test, and preferences for screening locations (home, clinic, or other)
- Education outcomes: changes in sexual health literacy (knowledge) about cervical cancer risk factors and screening (overall and by gender) and changes in attitudes (gendered stigma) toward cervical cancer risk factors and screening (overall and by gender)
- Cost-benefit analysis: net present value methodology will be used, which involves estimating the difference between the total discounted benefits and the total discounted costs for each of the cervical screening modalities (ie, HPV self-sampling vs VIA and Pap test)

### Mixed Methods of Data Collection

Informed consent will be obtained prior to data collection. Data will be gathered by using both quantitative and qualitative tools, which include interviewer-administered questionnaires, interviews, and focus groups.

#### Before and After SHE Sessions

All female and male participants will complete an interviewer-administered questionnaire before and after the SHE sessions. The questionnaire will include items on social demographics, medical histories, the stigmas surrounding and attitudes toward cervical cancer and screening, cervical cancer knowledge, screening practices, and relevant gender-specific questions (eg, those about menstruation and pregnancies). In addition, the women will be asked whether they would undergo cervical cancer screening and, if so, which of the methods of screening (VIA and Pap test or self-sampling) would they undergo and why. The men will be asked similar questions about whether they would encourage their female loved ones (mothers, sisters, cousins, or daughters) and about their preferred method of screening. Notes will be taken during the question and answer period in the SHE sessions to aid our understanding of participants’ knowledge, attitudes, and perspectives.

#### Cervical Cancer Screening Uptake and Results

Data will be collected about the number of women screened (through self-sampling vs through VIA or a Pap test), the number of HPV-positive women detected by each screening method, the number of HPV-positive women that followed up by undergoing a Pap test, and the number of Pap-tested women referred to colposcopy (follow-up).

#### Focus Group Consultations

We will engage women in 6 focus groups—4 with women from cohort A and 2 with women from cohort B. We will compile a list of female participants who express an interest in being contacted for the focus groups. We will then randomly select and invite women from each cohort until we recruit 8 to 10 women per focus group. Focus groups with women from cohort A will explore their experiences with, barriers to, and facilitators of using HPV self-sampling and suggestions for improving access and uptake. Further, 1 of the 4 focus groups will be for women with positive HPV results and will explore their care trajectory. Focus groups with women from cohort B will explore barriers and facilitators to the use of the kit and attitudes toward screening. One-on-one interviews will be arranged for women who have concerns about participating in focus groups. In addition, we will hold 2 focus groups (8-10 participants per focus group) with male participants to elicit their views about the project intervention and explore their perceived barriers and facilitators to the undertaking of cervical cancer screening by their female family members. These two focus groups will be held with health care providers to ascertain male participants’ views about HPV self-sampling and recommendations for practice and policy. All focus groups will last for approximately 90 minutes; will be facilitated by at least 2 members of the research team; and will be audio-recorded, transcribed, and translated to English by trained bilingual team members.

### Data Analysis Planning

Quantitative data analyses will be conducted by using SPSS 26 (IBM Corporation). Univariate descriptive statistics will be used to profile the study participants based on their survey responses. Inferential statistics, including dependent and independent 2-tailed *t* tests, 1-way ANOVA tests, and chi-square tests, will be performed to assess mean score differences for each outcome measure (eg, cervical knowledge, attitudes, and STI stigma) across and within study cohorts. Furthermore, bivariate and multivariate analyses, if feasible based on the sample size, will be conducted to determine the variables associated with HPV self-sampling uptake and predictors of screening practices.

Audio-recorded focus group interviews will be transcribed verbatim in Hindi and Marathi and translated into English. Each translated transcript will be reviewed and verified by at least 2 bilingual research team members to ensure cultural equivalence. A computer software program (NVivo; QSR International) will be used to aid with data management. For the data analysis, both inductive and deductive analyses will be applied to manually look for broad categories that are indigenous (articulated by the participants) and sensitizing (drawn from pre-existing theories and concepts) in the transcripts [52]. We will begin with the development of a coding framework that is informed by participants’ narratives, the research questions, and the RE-AIM framework. We will use a systematic approach that involves (1) familiarizing ourselves with the data; (2) generating initial codes; (3) developing a coding tree to guide the coding of transcripts; (4) identifying themes; (5) reviewing, defining, and naming themes; (6) analyzing and interpreting the narratives; and (7) producing reports and relevant documents based on the results. In addition, we will apply the triangulation of methods approach to a systematic comparison to verify study findings, elucidate complementary aspects of phenomena, and examine points of convergence and divergence for data that are specific to the different processes and outcomes of implementation.

### Ethics Approval

The study protocol received full ethical approval from the research ethics boards of Toronto Metropolitan University (formerly known as Ryerson University; reference number: REB 2020-104) and Tata Memorial Center (reference number: OIEC/3786/2021 /00003) in June 2021 and August 2021, respectively.

## Results

The anticipated short-term outcomes of our project are (1) increased HPV self-sampling among UNS women; (2) increased awareness and knowledge of HPV and cervical cancer among women and the men in their families; (3) increased sexual health literacy and knowledge among women and men in the three villages; (4) increased capacity among local community health professionals, health workers, and graduate students to carry out similar initiatives; (5) evidence of changes in gender norms to support gender and health equity; and (6) new available evidence for informing local and regional public health policies about how to advance HPV and cervical cancer screening in rural India.

The Preventing Cervical Cancer in India Through Self-Sampling study was funded in January 2020 for 15 months. Due to the COVID-19 pandemic, the project was extended by 1 year.

## Discussion

The anticipated main findings of our project are an increase in women’s uptake of cervical cancer screening and a higher propensity for using HPV-self sampling, which would corroborate earlier research on the feasibility and acceptability of self-sampling among low-income and marginalized women [17-31]. The project will also promote awareness, sexual health literacy, and the reduction of stigma among the studied communities by targeting both men and women. The engagement, partnership, and support of the male population in these communities will be critical in promoting women’s sustained uptake of cancer screening. The project will build capacity within low-income villages in India by training female community members and female health workers to educate women about cervical cancer and the use of HPV self-sampling. All of the sexual health educational resources that will be developed for this study, which will include storyboards, infographics, and videos, can be used with other communities across India that face this health challenge. Furthermore, our local collaborators have established strong connections with local government health agencies. Early on in the project, our local project coordinators will communicate with local government health agencies, which were identified by our local partners, and keep them informed about the study and its progress. The findings will be presented to local government agencies by our local partners. The proposed project, with its focus on women’s health, could result in the implementation of a high-value cervical screening program in other countries and contexts.

The evidence gained from the implementation of HPV self-sampling, the promotion of sexual health literacy, and the cost-benefit analysis will be used to advance public health policies and inform the scale-up of similar initiatives in other villages and states across rural India. Our project has the potential to impact thousands of women in India and other low-income countries.

## Abbreviations

HPV: human papillomavirus
PAC: project advisory committee
Pap: Papanicolaou
RE-AIM: Reach, Effectiveness, Adoption, Implementation, and Maintenance
SHE: sexual health education
STI: sexually transmitted infection
UNS: underscreened and never-screened
VIA: visual inspection with acetic acid
